# Childhood trauma influences the age of onset and severity of major depressive disorder via brain function

**DOI:** 10.1192/j.eurpsy.2021.876

**Published:** 2021-08-13

**Authors:** S. Chen, Y. Yuan

**Affiliations:** Department Of Psychosomatics And Psychiatry, ZhongDa Hospital, School of Medicine, Southeast University, Nanjing, China

**Keywords:** major depressive disorder, childhood trauma, brain function, social support

## Abstract

**Introduction:**

Associations between childhood trauma (CT), social support (SS), brain functions and major depressive disorder (MDD) is unknown.

**Objectives:**

This study aimed to investigate whether brain functions mediated associations between CT, SS, and MDD.

**Methods:**

164 MDD and 98 healthy controls (HC) were recruited and measured by HAMD-24 and HAMA. Some completed CT questionnaire (CTQ) and social support rating scale (SSRS). We examined amplitude of low-frequency fluctuation (ALFF) between the two groups and correlations between HAMD-24, HAMA and ALFF in MDD. Then, the peak voxels of the ALFF changed regions were used as seeds to analyze whole-brain functional connectivity (FC). Next, correlations between FC and clinical variables of MDD were performed. Last, mediation analysis was used to further determine whether ALFF or FC could mediate the associations between CT, SS, and different clinical variables in MDD patients.

**Results:**

Compared to HC, MDD showed decreased ALFF in right posterior cingulate (PCC_R), left postcentral gyrus, right precentral gyrus, and left thalamus (THA_L), but increased ALFF in right medial frontal gyrus, left subgenual anterior cingulate, and left middle occipital gyrus as well as decreased FC in bilateral PCC and THA_R. HAMD-24 had negative correlation with ALFF of THA_L, while positive with sexual abuse (SA) score in MDD. Mediation analysis revealed that FC of PCC_R mediated association between SA and baseline HAMD-24, and itself or together with SS mediated association between CT and onset age of MDD.

**Conclusions:**

CT may influence the depression severity and onset age of MDD by moderating FC of PCC_R only or together with SS.

